# Renal Stones and Gallstones Correlated with the Ten-Year Risk Estimation of Atherosclerotic Cardiovascular Disease Based on the Pooled Cohort Risk Assessment of Males Aged 40–79

**DOI:** 10.3390/jcm12062309

**Published:** 2023-03-16

**Authors:** Hui-Yu Chen, Chih-Jen Chang, Yi-Ching Yang, Feng-Hwa Lu, Zih-Jie Sun, Jin-Shang Wu

**Affiliations:** 1 Department of Family Medicine, National Cheng Kung University Hospital, College of Medicine, National Cheng Kung University, Tainan 704302, Taiwan; 2 The Department of Family Medicine, College of Medicine, National Cheng Kung University, Tainan 704302, Taiwan; 3 Department of Family Medicine, Ditmanson Medical Foundation Chia-Yi Christian Hospital, Chiayi 600566, Taiwan; 4 Department of Family Medicine, National Cheng Kung University Hospital, Dou-Liou Branch, College of Medicine, National Cheng Kung University, Yunlin 640003, Taiwan

**Keywords:** cardiovascular risk, renal stone, gallstone, male

## Abstract

Background: The risk of developing atherosclerotic cardiovascular disease (ASCVD) is unknown for subjects with both gallstones and renal stones, nor is it known whether there is a difference in the risk between gallstones and renal stones. This study aimed to determine the risk relationship between gallstones and renal stones and the risk of ASCVD in a male population. Methods: We recruited 6371 eligible males aged 40 to 79 years old who did not have a documented ASCVD history. The ten-year ASCVD risk was calculated using the pooled cohort equations developed by the American College of Cardiology (ACC) and the American Heart Association (AHA). The ASCVD risk score was classified as a low risk (<7.5%), an intermediate risk (7.5% to 19.9%), or a high risk (≥20%). The diagnosis of gallstones and renal stones was established based on the results of abdominal sonography. Results: Both gallstones and renal stones were associated with a high level of intermediate risk (OR = 3.21, 95% CI = 1.89–5.49, *p* < 0.001) and high risk (OR = 3.01, 95% CI = 1.48–6.12, *p* < 0.001), compared to individuals with no stones at all, after adjusting for the effects of other clinical variables. The possession of gallstones was associated with a higher level of high ASCVD risk (OR = 1.84, 95% CI = 1.31–2.59, *p* < 0.05) than that of renal stones. Conclusions: The ASCVD risk was higher for males with gallstones than for those with renal stones. Men with both types of stones faced a risk of ASCVD that was three times higher than that of men without stones.

## 1. Introduction

Renal stone disease and gallstone disease are commonly encountered in clinical practice. The prevalence of renal stones varies between 2% and 20% of the population, while that of gallstones has been reported to be in the range of 10–15% [1,2]. Previous research has suggested that 90% of renal stones are calcareous stones [3], while gallstones are categorized as either cholesterol stones (80–90%) or pigment stones (10–20%) [4]. Although the mechanisms underlying their development remain unclear, renal stones and gallstones share a number of risk factors, such as obesity, hypertension, diabetes, metabolic syndrome, and dyslipidemia [4,5].

Cardiovascular diseases (CVDs) cause significant morbidity and mortality, leading to around 18 million deaths per year worldwide [6]. Although both genders face the same lifetime risk of CVDs, males generally develop CVDs at a younger age [7]. Regarding CVD prevention, the guidelines of the American College of Cardiology (ACC) and the American Heart Association (AHA) recommend the use of a ten-year atherosclerotic cardiovascular disease (ASCVD) risk estimation [8]. Studies have indicated that the presence of both gallstones [9,10,11] and renal stones [12,13,14] is associated with an increased risk of CVD [9,10,11,12,13,14]. However, to our knowledge, two questions remain unanswered: what is the risk of CVD for males with both gallstones and renal stones? Additionally, is there a difference in CVD risk between gallstones and renal stones? Therefore, the aim of this study was to assess the ASCVD risk across different groups of males: males who had no stones, those with either gallstones or renal stones, and those with both types of stones.

## 2. Methods

To carry out this cross-sectional study, we first recruited males of 18 years of age or older who received given check-ups at the health examination center of the National Cheng Kung University Hospital during the period from June 2001 to August 2009. We then excluded individuals who were aged under 40 years old and over 79 years old (n = 3400), those who had a history of stroke or ischemic heart disease (n = 108), those who were deemed to be heavy drinkers, defined as an alcohol consumption of 196 gm/week (n = 47) [15], and those whose abdominal sonography exam showed signs of post-cholecystectomy syndrome (n = 106). In the final analysis, 6371 people were eligible to participate in the study. The study was approved by the Ethical Committee for Human Research of the National Cheng Kung University Hospital (IRB number: A-ER-111-366).

First, the participants were asked to fill out a questionnaire that included information about their personal medical history, as well as habits such as smoking, alcohol consumption, and regular exercise. The formula used for determining the body mass index (BMI) was weight (in kilograms) divided by height (in meters) squared. The behaviors of cigarette smoking and alcohol consumption were scored as currently exhibited and not currently exhibited, and exercise performed regularly was defined as more than 20 min of physical activity performed at least three times a week. The participants were then placed at rest in a supine position for at least 5 min, after which their systolic blood pressure (SBP) and diastolic blood pressure (DBP) were measured. They were considered to have hypertension if they either exhibited a blood pressure measurement of SBP/DBP ≥ 140/90 mmHg or had a positive history of hypertension. All participants were required to fast overnight for 10 h in order to ascertain their levels of glucose, hemoglobin A1c, cholesterol, triglyceride, and high-density lipoprotein cholesterol (HDL-C). An oral glucose tolerance test (75 gm) was performed with subjects who had no history of diabetes. In addition to some participants having a positive history of diabetes, the existence of diabetes mellitus was determined on the basis of a fasting plasma glucose level of ≥7 mmol/L, a 2 h post-load glucose level of ≥11.1 mmol/L, or a hemoglobin A1c level of ≥6.5%.

After fasting, all participants underwent abdominal sonography carried out by experienced radiologists who were unaware of this study. The ultrasounds were performed using a convex-type real-time electronic scanner (XarioSSA660A, Toshiba, Tokyo, Japan), with the results used as the basis for the diagnosis of renal stones and gallstones. A diagnosis of renal stones was established when the sonogram showed either (1) light points or light regions in the kidneys accompanied by vertical acoustic shadows or (2) several echo light bands, strong echoes, or acoustic shadows [16]. Sonographic findings of highly reflective echogenic foci in the gallbladder lumen were the basis of a diagnosis of gallstones [17]. We classified the participants according to their results for the sonography test, as individuals who had no stones, those with renal stones, those with gallstones, and those with both types of stones.

We calculated the ten-year ASCVD risk using the pooled cohort equations (PCE) designed by the ACC and the AHA [8]. The calculation of the ASCVD risk was based on age, sex, levels of cholesterol and HDL-C, chronic hypertension, diabetes, and smoking habits. The ten-year ASCVD risk scores were categorized as low risk (<7.5%), intermediate risk (7.5% to 19.9%), and high risk (≥20%).

### Statistical Analyses

We used the 20th version of the SPSS software (Chicago, IL, USA) to perform the statistical analyses. The continuous variables were expressed as means ± standard deviation, and the categorical variables were expressed as percentages. One-way ANOVA and the chi-square test were used to compare the continuous variables and the categorical variables, respectively, between the groups established on the basis of the presence or absence of gallstones and renal stones. Multinomial logistic regression was used to adjust for the effects of BMI, alcohol consumption, and regular exercise. The adjusted odds ratio (OR) and the 95% confidence interval (CI) were determined in order to assess the associations between the presence or absence of stones and the intermediate level and the high level, respectively, of ASCVD risk.

## 3. Results

The ten-year ASCVD risk score for the 6371 eligible males, with an average age of 52.9 ± 8.9 years old, was 9.5 ± 9.7%. In terms of their risk levels, 3633 participants (57%) fell into the low-risk category, 2006 (31.5%) were in the intermediate-risk category, and 732 (11.5%) were in the high-risk category. Table 1 summarizes their clinical characteristics according to the presence or absence of stones as none, only gallstones, only renal stones, and both gallstones and renal stones. Significant differences in age, SBP/DBP, history of hypertension and diabetes, serum HbA1C, cholesterol, and ten-year ASCVD risk were found between the groups. The rates of the prevalence of intermediate and high levels of ASCVD risk were 29.8% and 10.6%, respectively, for the group without stones, 37.6% and 19.4% for the group with gallstones, 36.9% and 12.5% for the group with renal stones, and 52.1% and 16.9% for the group with both types of stones.

Table 2 presents the factors that were independently associated with both intermediate and high levels of ASCVD risk. After adjusting for the effects of BMI, current alcohol consumption, and regular exercise (≥3 times/week) so that these characteristics of the subjects without stones could be used as benchmarks, the ASCVD risk was found to be higher among subjects with both types of stones (intermediate risk: OR = 3.21, 95% CI = 1.88–5.49, *p* < 0.001; high risk: OR = 3.01, 95% CI = 1.48– 6.12, *p* < 0.001), renal stones (intermediate risk: OR = 1.45, 95% CI = 1.23–1.70, *p* < 0.001; high risk: OR = 1.37, 95% CI = 1.09–1.73, *p* < 0.01), and gallstones (intermediate risk: OR = 1.78, 95% CI = 1.42–2.24, *p* < 0.001; high risk: OR = 2.53, 95% CI = 1.91–3.35, *p* < 0.001).

We compared the ASCVD risk between subjects with gallstones and those with renal stones after the adjustments for BMI, current alcohol consumption, and regular exercise (≥3 times/week) (see Table 3). The presence of gallstones was associated with a significantly higher level of high ASCVD risk (OR = 1.84, 95% CI = 1.31–2.59, *p* < 0.05) compared to the association of the same risk associated level with the possession of renal stones alone, but such a difference was not observed for the intermediate level of risk. On the other hand, subjects with both types of stones faced significantly higher levels of intermediate and high ASCVD risk compared to subjects with only renal stones (intermediate risk: OR = 2.22, 95% CI = 1.28–3.86, *p* < 0.01; high risk: OR = 2.19, 95% CI = 1.05–4.57, *p* < 0.05). Finally, subjects with both types of stones faced a significantly higher level of intermediate ASCVD risk compared to subjects with only gallstones (intermediate risk: OR = 1.80, 95% CI = 1.01–3.20, *p* < 0.05).

## 4. Discussion

In this study, the intermediate level (OR = 3.21) and high level (OR = 3.01) of ASCVD risk were three times higher for males with both gallstones and renal stones compared to individuals with no stones. The presence of both types of stones was also associated with a higher intermediate level of ASCVD risk, with OR values that were 1.7 times and 2.2 times higher than the values for the presence of gallstones alone and renal stones alone, respectively. In addition, the high level of ASCVD risk was 84% higher for subjects with only gallstones compared to those with only renal stones.

The association of gallstones and renal stones with the risk of CVD found in this study is consistent with the findings of other studies conducted on individuals with either gallstones [9,10,11,18,19,20] or renal stones [12,13,14]. More specifically, a recent meta-analysis conducted by Zhao et al. suggested that the risk of incidence (HR = 1.24) and prevalence (OR = 1.23) of CVD were higher among people with gallstones [9]. In addition, renal stone diseases have been associated with a high risk of subsequently experiencing cardiovascular events [12,14,21,22,23,24,25]. In another meta-analysis conducted by Liu et al., it was reported that the presence of renal stones increased the risk of developing myocardial infarction by 29% (HR = 1.29) and increased the risk of stroke by 40% (HR = 1.40) over a follow-up period of 5 to 11 years [14]. However, none of these studies compared the cardiovascular risk associated with the presence or absence of gallstones and renal stones, nor did they specify the intermediate and high levels of cardiovascular risk. To our knowledge, our study is the first to take into consideration both the intermediate and high risks of CVD among males with both gallstones and renal stones while also comparing the associations of ASCVD with the possession of renal stones and with the possession of gallstones.

The association of the presence of gallstones and renal stones and with CVD can be explained based on the common pathophysiology of these conditions, even though the underlying mechanisms are not fully understood [9,24,26]. Gallstones and renal stones share common risk factors such as age, obesity, chronic hypertension, and diabetes mellitus [9,24,26]. There exists a number of possible mechanisms, such as inflammation, insulin resistance, and calcium deposits, that might explain the relationship between both types of stone diseases and an increased risk of CVD. Studies have shown that aberrant inflammation and oxidative stress are involved in the development of renal stones, gallstones, and CVD because they play significant roles in metabolic syndromes, obesity, and insulin resistance [9,26,27,28]. It has also been suggested that insulin resistance and the inflammatory response in the gallbladder and kidneys promote atherosclerosis and vasculopathy in the blood vessels [9,24,27,28]. In addition, insulin resistance has been identified as a determining factor in the formation of gallstones, because it leads to the excess secretion of biliary cholesterol and gallbladder dysmotility [29].

Insulin resistance has also been associated with the formation of renal stones as a result of the increasing deposition of calcium oxalate crystals and decreasing urine pH [5]. In general, calcium has been shown to play an important role in many physiological processes, including blood coagulation, muscle contraction, nerve conduction, and epithelial secretion and absorption [30]. More specifically, it has been suggested that the precipitation of calcium salts is the main factor promoting the formation of renal stones [5] and elevated concentrations of calcium might increase vascular calcification and blood coagulation, which are considered to be factors involved in the development of CVD [31]. In addition, the deposition of calcium salts is important for the formation of pigment gallstones and is a nidus of the development of cholesterol gallstones [30]. It should be noted that it is as yet unknown why subjects with gallstones face a higher cardiovascular risk than those with renal stones. Bile acids are important elements of the diversity and metabolic activity of the microbiota. Additionally, the bile acid pool is conducted by the gut microbiota [32]. Recent research suggests that a disturbance of the secretion of bile acids contributes to the formation of gallstones and dysbiosis of the gut microbiota, the latter of which has emerged as a novel CVD risk [33,34]. Specifically, dysbiosis has been implicated in CVD, together with various aspects of cardiometabolic syndrome: obesity, hypertension, chronic kidney disease, and diabetes. A mechanistic link between the gut microbiota formation of trimethylamine-N-oxide (TMAO) and CVD has been demonstrated [35]. Therefore, it might be possible to partly explain the higher cardiovascular risk associated with gallstones on the basis of the metabolic function of the gut microbiota.

In addition to the clinical variables included in the estimation of the ten-year ASCVD risk, such as age and sex, as well as the levels of total cholesterol and of HDL-C and the existence of chronic hypertension, diabetes, and smoking, this study also revealed a relationship between BMI and the risk of ASCVD. A number of studies have shown that BMI [36] is positively related to the future development of CVD, while alcohol consumption [37] and regular exercise [38] have been shown to have an inverse relationship. In the current study, we found that BMI was positively associated with both intermediate and high levels of ASCVD risk, an observation which is compatible with the findings of other studies. [36]

In this study, after excluding 47 participants who were deemed to be heavy drinkers, all the remaining drinkers reported low to moderate levels of alcohol consumption. In the final analysis, a negative correlation was found between alcohol consumption and an intermediate level of ASCVD risk. This result is consistent with the findings of previous studies, demonstrating that low to moderate levels of alcohol consumption are associated with decreased cardiovascular morbidity and mortality [37]. As for the lack of a significant correlation between low to moderate levels of alcohol consumption and a high level of ASCVD risk, the explanation may be that the impact of these levels of alcohol consumption is relatively weak compared to the impacts of the traditional risk factors for CVD, but further research is required to determine whether or not this assumption is correct. It has been suggested that regular physical activity may be beneficial in either directly or indirectly reducing the risk of CVD [38]. However, to our surprise, we found a positive relationship between regular exercise and an intermediate level of ASCVD risk, although the correlation between regular exercise and a high level of ASCVD risk was insignificant. These findings might be a result of the possibility that people started to perform regular exercise after becoming aware of their cardiovascular risk factors, such as elevated levels of blood pressure, blood glucose, or cholesterol, in this cross-sectional study. In Taiwan, a national preventive health screening program for adults of ≥40 years of age, similar to the health check program carried out by the NHS (National Health Service) in England for adults aged 40–74 years of age, was implemented in 1996. Such findings might provide more opportunities to raise people’s awareness of the benefits of physical activity.

There are several limitations of this retrospective cross-sectional study. Firstly, we were unable to make any causal inferences regarding the relationship between the presence of renal stones or gallstones and the risk of ASCVD. To address this shortcoming, prospective research should be carried out as a means of clarifying the cause-and-effect relationships which might be at play. Secondly, since the study involved only males aged 40 to 79 years old, the finding of a relationship between the possession of renal stones and gallstones and the development of CVD cannot be generalized to the entire population. Thirdly, a wide range of sensitivities and specificities have been reported in regard to ultrasonography, probably owing to variations in technique, body habitus, patient population, or interference from bowel gas [39]. In this study, all the abdominal sonographies were carried out by a fixed number of experienced radiologists. Additionally, we could not identify the exact sizes or the specific subtypes of renal stones [40] and gallstones [41] affecting each subject, and the processes through which different sizes and different subtypes form might lead to different cardiovascular risks. Further research may be needed to examine the associations between various subtypes of stones and the risk of CVD. Additionally, although we adjusted for body mass index, alcohol consumption, and exercise to reduce the degree of bias, the influences of diet, medication, and genetics, which were not collected in this study, cannot be completely ruled out. Finally, some studies have reported that the use of pooled cohort equations to estimate ASCVD risk appears to lead to the overestimation of this risk in Asian populations [42]. However, despite the lack of a validated score for an Asian population, the pooled cohort approach remains an option for clinicians who wish to discuss possible strategies for the prevention of CVD with their patients.

## 5. Conclusions

Both gallstones and renal stones were associated with high levels of intermediate- and high-level ASCVD risk. The risk was higher in males with gallstones than in those with renal stones. Males with both types of stones faced a three-times-higher risk compared to individuals who had no stones at all, and they also faced a higher risk compared to males who had only one type of stone. In clinical practice, cardiovascular risk assessment should be considered for individuals with gallstones, while it cannot be ignored in the case of individuals with renal stones.

## Figures and Tables

**Table 1:** Comparison of clinical parameters between males classified according to the possession of renal stones and gallstones.

	Non-Stone (n = 5052)	Gallstone (n = 407)	Renal Stone (n = 841)	Both Stones (n = 71)	*p*-Value
Age, year	52.4 ± 8.8	56.4 ± 10.0	53.5 ± 8.7	58.0 ± 9.7	<0.001
Body mass index, kg/m^2^	25.1 ± 3.1	25.2 ± 2.9	25.4 ± 2.9	25.7 ± 2.7	0.06
Systolic BP, mmHg	123 ± 17	127 ± 19	125 ± 18	129 ± 17	<0.001
Diastolic BP, mmHg	75 ± 11	76 ± 11	77 ± 11	79 ± 12	<0.001
Hypertension	892 (17.7)	117 (28.7)	228 (27.1)	28 (39.4)	<0.001
Diabetes mellitus	419 (8.3)	57 (14)	94 (11.2)	11 (15.5)	<0.001
HbA1c, %	5.9 ± 1.1	6.1 ± 1.3	6.0 ± 1.1	6.0 ± 1.2	0.002
HDL-C, mmol/L	1.15 ± 0.30	1.14 ± 0.29	1.16 ± 0.31	1.15 ± 0.31	0.646
Cholesterol, mmol/L	5.15 ± 0.96	5.17 ± 0.98	5.25 ± 0.93	5.07 ± 1.03	0.032
Triglyceride, mmol/L	1.69 ± 1.05	1.59 ± 0.98	1.75 ± 1.13	1.68 ± 0.99	0.534
Current alcohol consumption	965 (19.1)	60 (14.7)	146 (17.4)	15 (21.1)	0.112
Current smoking	1540 (30.5)	122 (30.0)	245 (29.1)	15 (21.1)	0.330
Regular exercise ≥ 3/week	563 (11.1)	45 (11.1)	87 (10.3)	5 (7.0)	0.656
Calculated ASCVD risk,					
Low	3010 (59.6)	175 (43.0)	426 (50.7)	22 (31.0)	<0.001
Intermediate	1506 (29.8)	153 (37.6)	310 (36.9)	37 (52.1)	
High	536 (10.6)	79 (19.4)	105 (12.5)	12 (16.9)	

Data expressed as mean ± standard deviations or number (%). BP, blood pressure.

**Table 2:** Multinomial logistic regression models for the associations of stone status with intermediate and high levels of ASCVD risk, with a non-stone group for reference.

	Intermediate vs. Low Risk OR (95% CI)	High vs. Low Risk OR (95% CI)
Body mass index, kg/m^2^	1.086 (1.066–1.106) ***	1.028 (1.001–1.056) *
Current alcohol consumption, yes vs. no	0.647 (0.564–0.743) ***	1.019 (0.820–1.267)
Exercise ≥ 3/week, yes vs. no	1.294 (1.079–1.552) **	1.152 (0.888–1.494)
Renal stone vs. non-stone	1.445 (1.230–1.696) ***	1.374 (1.089–1.734) **
Gallstone vs. non-stone	1.784 (1.421–2.241) ***	2.529 (1.909–3.351) ***
Both stones vs. non-stone	3.214 (1.881–5.490) ***	3.007 (1.479–6.115) **

* *p* < 0.05, ** *p* < 0.01, *** *p* < 0.001.

**Table 3:** Multinomial logistic regression models for the association of stone status with intermediate and high levels of ASCVD risk, with a renal stone group and gallstone group, respectively, for reference.

	Intermediate vs. Low Risk OR (95% CI)	High vs. Low Risk OR (95% CI)
Reference of renal stone		
Non-stone vs. renal stone	0.692 (0.590–0.813) ***	0.728 (0.577–0.919) **
Gallstone vs. renal stone	1.235 (0.949–1.252)	1.841 (1.309–2.589) ***
Both stones vs. renal stone	2.224 (1.281–3.863) **	2.189 (1.049–4.566) *
Reference of gallstone		
Non-stone vs. gallstone	0.560 (0.446–0.704) ***	0.395 (0.298–0.524) ***
Renal stone vs. gallstone	0.810 (0.622–1.054)	0.543 (0.386–0.764) ***
Both stones vs. gallstone	1.801 (1.013–3.201) *	1.189 (0.560–0.764)

Adjustment for BMI, alcohol consumption, and regular exercise. * *p* < 0.05, ** *p* < 0.01, *** *p* < 0.001.

## Data Availability

Health examination center of the National Cheng Kung University Hospital during the period from June 2001 to August 2009.
